# Epidemiology and Treatment of Distal Radius Fractures in Finland—A Nationwide Register Study

**DOI:** 10.3390/jcm11102851

**Published:** 2022-05-18

**Authors:** Leena Raudasoja, Samuli Aspinen, Heidi Vastamäki, Jorma Ryhänen, Sina Hulkkonen

**Affiliations:** 1 Department of Hand Surgery, Helsinki University Hospital, University of Helsinki, 00029 Helsinki, Finland; samuli.aspinen@hus.fi (S.A.); jorma.ryhanen@hus.fi (J.R.); sina.hulkkonen@helsinki.fi (S.H.); 2 Sports Trauma Research Unit, Hospital Mehiläinen Neo, 20520 Turku, Finland; heidi.vastamaki@fimnet.fi

**Keywords:** distal radius fracture, distal forearm fracture, wrist fracture, epidemiology, incidence, age, surgical procedures, register study, Finnish health care register

## Abstract

A distal radius fracture (DRF) is one of the most common fractures in emergency units, the treatment of which requires considerable health care resources. We analyzed the incidence rate for DRFs and the incidence rate of operative treatment over a five-year period, 2015–2019, for the entire population of Finland and all ages. Data was obtained from the Finnish National Care Register for Health Care. The results were counted as cases per 100,000 person/years and standardized with the European Standard Population 2013. The mean annual incidence rate of DRF was 204.90 (203.21–206.59) in specialist care and 69.53 (68.55–70.52) in primary care. It peaked among the pediatric population and among elderly women, in whom it was more than four times as common compared to men of the same age. No increase in the incidence rate of DRFs was found. The mean incidence rate of operative treatment was 45.66 (45.66–45.66)/100,000 person/years, 2015–2019; women were more likely to undergo operative treatment. Altogether, 15–18% of DRFs were operated on over the study period. The annual incidence rate of operations seemed to plateau compared to earlier studies in Finland.

## 1. Introduction

Distal radius fractures (DRF) are the most common of all fractures encountered both in hospital emergency care units, as well as in outpatient duty offices [1]. A clear bimodal incidence has been described, with children under 15 years and adults over 50 years of age at the greatest risk of fracture [2,3,4]. In the elderly group, osteoporosis has proven to be the main risk factor for fracture [1]. There are several epidemiological studies exploring the incidence of DRFs; however, most of the epidemiological data are based on evaluations extrapolated from limited city or hospital district data [4,5,6,7,8]. Only a few previous papers have reported nationwide incidence rates of DRFs [9,10,11,12,13]. In Sweden, Rundgren et al. obtained data from the Swedish Fracture Register, as did Bergh et al. [10,14]. Südow and Navarro used the Swedish National Patient Register for pediatric data [11]. In The Netherlands, de Putter et al. contributed a population-based study using the Dutch National Injury Surveillance System and the National Hospital Discharge Registry [12]. In Korea, Jo et al. [13] analyzed a nationwide database acquired from the Korean Health Insurance Review Service Registry (which covers 97% of the population). Globally, in Asian countries, the incidence of DRFs is lower compared to those in Northern Europe and America [15]. Some publications have predicted that the incidence of DRFs will increase in the future due to the ageing of the population [16,17]

While closed reduction and cast immobilization is the treatment of choice in most DRFs, the number of fractures that are treated operatively increased rapidly in the early 21st century, most likely due to the introduction of volar locking plates to treat these fractures [18]. Several studies have reported this increase [9,13,18]. Since 2008, the rate of operative treatment for DRFs has plateaued in Finland [19]. At present, volar plating is the most common operative modality in developed countries [4,20,21], although regional differences might exist.

As emergency care may be fragmented and the coding of DRFs vary, the true incidence of DRFs may be underestimated in most studies. Therefore, a large unselected series is necessary to evaluate the true incidence of these fractures. Finland, a nation with approximately 5.5 million inhabitants, has the benefit of nationwide in- and outpatient registries. Thus, studying these registries gives us a unique view of DRF incidence and treatment patterns.

We wanted to investigate the nationwide incidence of DRFs and whether it increased or decreased over a five-year period. In addition, we wanted to explore the nationwide incidence and type of operative treatment for DRFs. In our study, we used a nationwide registry and standardized the incidences, thus making these more comparable to other countries, as the age distribution varies between countries.

## 2. Materials and Methods

The Care Register for Health Care, Specialist Care (former name: The Finnish National Hospital Discharge Register, FNHDR) is a statutory nationwide registry for public and private hospitals. Worldwide, it is one of the oldest individual-level hospital discharge registers covering an entire country [22]. The database contains comprehensive information on patient characteristics, diagnoses, and both in- and outpatient surgical procedures performed. Since 2011, the Register of Primary Health Care Visits has been collecting data on the availability and activities of outpatient primary health care. The data collection covers all outpatient primary health care delivered in Finland as part of the public health care. The coverage of outpatient cases of private sector institutions is still under development.

In this study, we combined and analyzed the Care Register for Health Care data of all DRFs and surgical procedures on both the pediatric and adult populations, in both primary and specialist care, who were registered during the time interval 1 January 2015–31 December 2019. The patients were identified using the International Classification of Diseases, tenth revision (ICD-10) diagnosis codes (S52.5, S52.6). Furthermore, we investigated the number and type of surgical procedures using the Nordic Medico-Statistical Committee (NOMESCO) classifications of Surgical Procedures (NCSP) for procedure codes NCJ62, NCJ64, NCJ70, NDJ62, NDJ64, NDJ70 (Supplementary Table S1). If a patient had multiple operation codes, open reduction and internal fixation ORIF (NCJ62/NDJ62) were considered the primary operation code to avoid multiplying the results. If the patient was recorded in both primary and specialist care data, data from the specialist care was handled primarily. Though the majority of DRFs are conservatively treated, they are covered by the Care Register for Health Care Specialist Care data, as emergency duty in Finland is predominantly centralized in hospitals, and only a small minority are registered primarily with the Care Register Primary Care without any visit to hospital outpatient clinic.

We only included incident cases of DRF in the study. As we used register data, we did not have information on the affected side or possible new treatment occasions due to the same fracture or a new one. This exclusion was to avoid multiplying the results.

We obtained data from the National Institute for Health and Welfare (Findata) according to the following list:

1. All registered DRFs with code 52.5 or 52.6 between 1 January 2015 and 31 December 2019;
2. Date of registration;
3. Age of the patient at the first registration date;
4. Only patients with Finnish social security number;
5. Procedure of treatment (cast or operation according to NOMESCO classification, Supplementary Table S1);
6. If the patient was registered in both the Specialist Care Register and the Primary Care Register, only the Specialist Care Register was utilized.

Access to data was granted after Findata (data permit authority operating at the National Institute for Health and Welfare) approved the study design (THL/5178/14.02.00/2020). The research was a register-based blinded analysis and did not include identifiable individual participants. Thus, no informed consent or ethical approval was sought. This is in accordance with Finnish legislation (Medical Research Act 488/1999, amendments 295/2004, 794/2010).

### Statistical Analysis

Over the study period, the mean and annual incidence rates of DRFs were counted as cases per 100,000 person/years by dividing the number of new cases in each age group by the population of that age group in Finland. The mean incidence rates for operations due to the distal radius fractures were calculated in a similar manner annually and in age groups of ten-year intervals from 0–9 years old, 10–19 yo, 20–29 yo, etc., up to 80 yo and above. All the analyses were run for men and women separately and combined. Standardization for the rates was carried out using the direct method with the European Standard Population 2013 divided into 5-year age groups as a standard. Finally, the 95% confidence intervals (CI) were estimated assuming the Poisson distribution of the cases. The analyses were run with RStudio software version 1.3.1073.

## 3. Results

### 3.1. Incidence Rate of DRF

During 2015–2019, 77,517 incident cases of DRFs in total were registered in the Care Register of Health Care. The mean annual standardized incidence rate (95% confidence interval) per 100,000 person/years of DRFs was 204.90 (203.21–206.59) in specialist care and 69.53 (68.55–70.52) in primary care; among men, 146.41 (144.37–148.40) in specialist care and 44.81 (43.68–45.93) in primary care, and among women, 254.56 (251.92–257.21) in specialist care and 90.31 (88.73–91.88) in primary care. The incidence rates are displayed in Table 1.

### 3.2. The Incidence of DRFs in Different Age Groups

**Figure 1:**
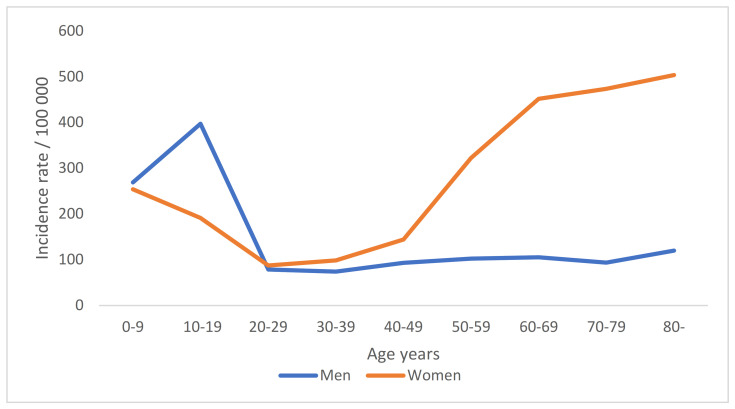
Mean annual standardized incidence rates of distal radius fractures (DRF) per 100,000 person/years by age groups, stratified by sex, 2015–2019.

In the Finnish population, the incidence rates of DRFs were followed a bimodal distribution with distinct peaks in the pediatric and elderly populations in 2015–2019. However, the incidence curve differed in men and women: while the incidence rate was higher among boys as children and teenagers in the 10–19 age group, after 60 years of age, the incidence among women was more than four-fold that among men. The age distribution of DRF incidence is presented in Table 2 and Figure 1.

### 3.3. The Annual Incidence of DRFs

Over the study period, the annual incidence rates of the DRFs did not change remarkably. The annual incidence rate of the DRF seemed to decline in primary care, whereas it increased in specialist care (Supplementary Table S2).

Among men, the incidence rates of the DRFs remained relatively stable over the study period. However, a trend of slightly increasing incidence rates of DRFs among the two youngest age groups was seen (Supplementary Table S3).

The annual incidence rates of the DRFs among women did not differ in the younger age groups, but from age 60 and over, there was a small increase in fractures (Supplementary Table S3). The annual age group incidence rate curves are available in Supplementary Figures S1 and S2.

### 3.4. Operative Treatment

During 2015–2019, 12,806 operatively treated DRFs were registered. The mean standardized incidence rate (95% CI) per 100,000 person/years for any type of operative treatment due to the DRF was 45.66 (45.66–45.66) over the study period, 31.14 (30.20–32.06) among men, and 58.58 (57.32–59.85) among women (Table 1). The annual incidence rates for the operative treatment were relatively stable, increasing slightly from 2015 to 2019 (Table 3 and Supplementary Table S4). Of all the DRFs diagnosed during 2015–2019 in Finland, 15–18% were operated on annually.

### 3.5. Operative Techniques

**Figure 2:**
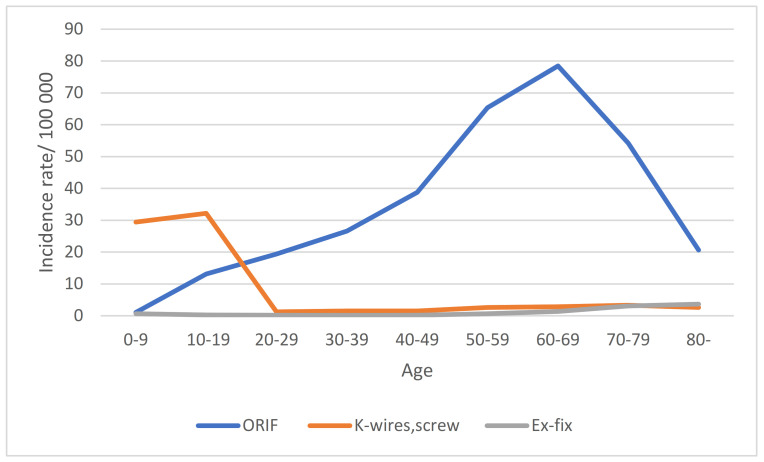
The mean annual incidence rates per 100,000 person/years of different operation types in age groups.

Over the study period, 80% of operations were open reductions and internal fixations (ORIF) with plates. K-wires were most often used among the pediatric population: the mean annual incidence rate (95% CI) per 100,000 person/years of K-wire fixation was 29.46 (29.46–29.46) in the 0–9 age group and 32.24 (32.23–32.24) in the 10–19 age group, while in the adult population, it was a mean 1.24–3.28. Among adults, ORIF was most commonly used, and external fixation was almost entirely abandoned. (Table 4 and Figure 2). In the elderly groups (70 yo and over), surgical activity decreased.

## 4. Discussion

The mean annual incidence rates (95% CI) of DRFs in our nationwide study of 27.6 million person/years of Finnish population, were 204.90 in specialist care and 69.53 in primary care per 100,000 person/years in 2015–2019. The incidence rate of the DRFs found in this study on a populational level is comparable to other Nordic register studies. In Sweden, Mellstrand-Navarro et al. [9] studied the Swedish National Patient Registry between 2005 and 2010: the incidence rate of the DRFs was found to be 31–33 per 10,000 person/years across the entire population of Sweden. Furthermore, Abrahamsen et al. [23] studied the Danish Hospital Discharge Register database and found that the age-adjusted incidence rate of distal forearm fractures was comparable to that of the Swedish population. Similar incidence rates have also been presented in Swedish, Norwegian, and Canadian regional studies [4,6,7,8], as well as in an American insurance-based study [3]. Our incidence rate was also similar to the Korean one; Jo et al. [24] analyzed a nationwide database acquired from the Korean Health Insurance Review and Assessment Service registry from 2011 to 2015. They found age-adjusted incidence of fractures to vary between 220 and 290/100,000 person/years.

Despite the similarities between the epidemiological studies of DRFs, the incidence rates they present are not directly comparable. Most studies are from a restricted area or have registered or studied only adults. Furthermore, the distinction between the distal forearm fracture and distal radius fracture is not always clear. In our register, we combined S52.5 and S52.6: S52.6 is often used when there is a distal ulnar styloid fracture. This combination is mostly used in the literature that uses ICD-10 coded databases. However, in the USA, for example, ICD-9 was the most widely used [3].

The overall incidence rate in the present study was relatively stable over the study period and no significant changes in the annual incidences were found. Most other published epidemiological studies have suggested that the incidence rate of DRF has stayed relatively stable over the past three decades. In Sweden, Mellstrand-Navarro showed that the incidence rate of DRF s in the whole population did not change but surgery increased from 5.82/10,000 to 7.44 during their five-year study period [9].

However, in our study, the incidence rate exhibited a bimodal distribution, with a peak among children and adolescents and a remarkable increase among women over 60 years of age. Even though the incidence rate of DRF has remained relatively stable, the bimodal distribution of these fractures is emphasized in this study. The number of elderly women (age 60 and above) and pediatric patients with DRFs increased over the study period. The same trend has been observed worldwide [25,26,27]. Though some annual incidence changes may be attributable to varying weather conditions between different years [8] and changing pastime activities among children [27], no straightforward explanation for the incidence increase among pediatric and elderly populations can be expressed.

The incidence of operations for DRFs showed a slightly increasing trend over our study period. Almost a fifth of all DRFs were operated on. The trends in the operations due to the DRFs were previously studied using the Finnish specialist healthcare register by Mattila et al. [18], showing a doubled share of operative treatment from 1998 to 2008. A similar trend of increasingly common operative treatment has been reported in several countries [4,9,20,28]. In Canada, during the years 2004–2013, 83% of DRF cases in the adult population were treated conservatively [6]. In the USA [28], the percentage of conservatively treated fractures decreased from 82% to 70% over the period 1997–2007. Since then, based on the results derived from the FNHDR data published by Hevonkorpi et al. [19] in 2009–2016, the rate of operative treatment for DRFs has remained constant in those over 50 years of age. While the rate of surgical treatment has generally plateaued in Finland, contradictory results for the level of surgical treatment can be found [21,29]. Jo et al. reported an increase in the incidence level of operated DRFs from 32.6 to 38.0% in Korea [24].

The development of volar locking plates has paved the way for anatomical reduction and stable internal fixation of even highly comminuted fractures. Thus, the decreased need for postoperative fracture support has led to faster rehabilitation. In our study, we assumed that most of the open reduction and internal fixation operations were performed with volar locking plates, which have become increasingly popular, while external fixation has been almost entirely abandoned. A similar trend can be seen overall in all Western countries [3,4,18,19]. In children, the use of K-wires is still the method of choice.

The strength of this study is that it is based on data from a national register: the Care Register for Health Care, including both in- and outpatient data from primary and specialist care from the entire country, with complete demographics of the background population. The specialist care data of the Care Register for Health Care (former Hospital Discharge Registry, FNHDR) was founded in 1969 and is mandatory for all hospitals in Finland, including all private and public hospitals. FNHDR has proven to have high validity [22] and has been widely used in epidemiological research. Though the majority of DRF are conservatively treated, they are covered by the specialist Care Register for Health Care, as emergency duty in Finland is predominantly centralized in hospitals. The Care Register for Health Care’s primary care register was established in 2011 to collect information besides hospital care and discharges more comprehensively. Even though it has not yet been validated, it allows for additional information and completes the data. According to data from the Finnish Institute for Health and Welfare, 87% of municipal health centers sent their data to the register in 2012. Since then, the coverage of the register has improved. Unfortunately, coverage of private sector medical centers outpatient clinics is still under development. Both registers are maintained by the Finnish Institute for Health and Welfare.

This epidemiological study has some limitations. First, bilateral fractures are not recorded, because the laterality of the fracture is not registered. Secondly, because of the nature of the data, it is impossible to distinguish between a new fracture and a new patient contact because of a previously suffered injury. Thus, possible new vs. refractures of a patient who has already suffered a DRF during the five-year period is not recorded. While not common, some underestimation may have occurred. Additionally, as mentioned earlier, DFRs treated only in private physicians’ offices as outpatients are not mandatorily registered, although operations from the private hospitals are. However, all the duplicates concerning primary care and specialist care were cleansed from the registry before analysis.

This being a register study, we did not obtain information on the fracture trauma mechanism, pathogenesis, or fracture classification, nor the exact type of surgery (volar/dorsal plate). Patient comorbidities, e.g., osteoporosis, diabetes, and hypertension, were collected from the database, but their analysis fell beyond the scope of this study. However, we plan to analyze the connection of diagnosed osteoporosis to DRFs.

## 5. Conclusions

The incidence rate of distal radius fractures in Finland is comparable to those of other countries and has remained stable during 2015–2019. While most fractures are treated nonoperatively, the number of operations showed a slight increasing trend. ORIF accounts for 80% of adult DRF operations, while external fixation has been almost entirely abandoned. K-wire fixation is still the method of choice in the pediatric population.

## Figures and Tables

**Table 1:** Standardized incidence rates (95% confidence intervals) of distal radius fractures (DRF) and operations due to DRFs per 100,000 person/years in Finland, 2015–2019.

Population	Primary Care	Specialist Care	Incidence of Operations
Men	44.81 (43.68–45.93)	146.41 (144.37–148.40)	31.14 (30.20–32.06)
Women	90.31 (88.73–91.88	254.56 (251.92–257.21)	58.58 (57.32–59.85)
All	69.53 (68.55–70.52)	204.90 (203.21–206.59)	45.66 (45.66–45.66)

**Table 2:** Age distribution of men and women incidence rates per 100 000 person/years with distal radius fracture (DRF) years 2015–2019.

Age Group, Years	Incidence Rate of DRFs Men	Incidence Rate of DRFs Women
0–9	269.06	253.97
10–19	397.23	191.32
20–29	78.9	87.5
30–39	74.1	98.39
40–49	93.12	144.27
50–59	102.53	322.62
60–69	105.23	451.81
70–79	93.63	473.66
80–	119.95	503.68

**Table 3:** Standardized annual incidence rates (95% confidence intervals) per 100,000 person/years of different types of operations due to distal radius fractures, 2015–2019.

Year	2015	2016	2017	2018	2019
Any operation	43.07 (41.33–44.80)	43.69 (41.95–45.44)	46.97 (45.17–48.78)	47.35 (45.54–45.16)	47.42 (45.60–49.23)
ORIF *	32.70 (31.18–34.21)	33.9 (32.39–35.47)	38.16 (36.53–39.79)	38.90 (37.25–40.54)	39.43 (37.78–41.09)
K-wires, screw **	9.07 (8.28–9.87)	8.89 (8.10–9.68)	7.91 (7.17–8.65)	7.82 (7.08–8.55)	7.72 (6.99–8.45)
Ex-fix ***	1.30 (0.99–1.62)	0.88 (0.63–1.12)	0.9 (0.65–1.15)	0.64 (0.43–0.85)	0.26 (0.13–0.39)

* Open reduction and internal fixation with a plate, wires, and screw; ** Fracture fixation with key-wires or screw; *** Fracture fixation with an external fixation device.

**Table 4:** Standardized incidence rate per 100,000 person/years of distal radius fracture surgery and different operation types in different age group years 2015–2019 with 95% confidence intervals.

Age	ORIF	K-Wires, Screw	Ex-Fix	Incidence Rate of Any Surgery Per Age Group
0–9	1.06 (1.06–1.06)	29.43 (29.43–29.43)	0.68 (0.07–0.07)	30.55 (30.55–30.55)
10–19	13.15 (13.15–13.15)	32.17 (32.17–32.17)	0.23 (0.23–0.23)	45.55 (45.55–45.55)
20–29	19.37 (19.37–19.37)	1.24 (1.24–1.24)	0.15 (0.15–0.15)	20.75 (20.75–20.75)
30–39	26.57 (26.57–26.57)	1.50 (1.50–1.50)	0.14 (0.14–0.14)	28.22 (28.21–28.22)
40–49	38.79 (38.79–38.79)	1.51 (1.51–1.51)	0.15 (0.15–0.15)	40.46 (40.45–40.46)
50–59	65.32 (62.32–65.32)	2.64 (2.64–2.64)	0.65 (0.65–0.65)	68.61 (68.61–68.61)
60–69	78.46 (78.45–78.46)	2.83 (2.83–2.83)	1.33 (1.33–1.33)	82.61 (82.61–82.61)
70–79	54.19 (54.19–54.19)	3.28 (3.28–3.28)	3.05 (3.05–3.05)	60.51 (60.51–60.51)
80–	20.66 (20.65–20.66)	2.63 (2.63–2.63)	3.65 (3.65–3.65)	26.93 (26.93–26.93)

## Data Availability

The data for this research was claimed from the National Institute for Health and Welfare, Helsinki, Finland. However, restrictions apply to the availability of this data, which was used under the license for the current study and is not publicly available. However, the authors may provide the data upon reasonable request and with the permission of the National Institute for Health and Welfare, Helsinki, Finland.

## Supplementary Materials

The following supporting information can be downloaded at https://www.mdpi.com/article/10.3390/jcm11102851/s1: Table S1: Types of surgical procedures for distal radius fractures according to NOMESCO classification. Table S2: Annual incidence of distal radius fractures (DRF) in specialist and primary care (with 95% confidence intervals). Table S3: Incidence of DRF men and women in different age groups and years. Table S4: Annual incidence of the DRF operative treatment in men and women. Figure S1. Annual incidence rate/100,000 person/years in distal radius fractures in men of different age groups for the years 2015–2019. Figure S2. Annual incidence rate/100,000 person/years in distal radius fractures in women of different age groups for the years 2015–2019.
