# Supplementary material for: Epidemiology and Treatment of Distal Radius Fractures in Finland—A Nationwide Register Study
Source: J Clin Med. 2022 May 18;11(10):2851. doi: 10.3390/jcm11102851 (PMC9143261; doi:10.3390/jcm11102851)
Supplement: Supplementary file 1 [file jcm-11-02851-s001.zip › jcm-1653341-supplementary.pdf]

Table S1. Types of surgical procedures for distal radius fracture according to NOMESCO classification.

NCJ 62	Internal fixation of fracture of forearm using plate
NCJ64	Other internal fixation of fracture of forearm (screw, pin)
NCJ70	External fixation of fracture of elbow or forearm, body of ulna and radius
NDJ62	Internal fixation of fracture of wrist or hand using plate and screws, schaphoid
NDJ64	Internal fixation of fracture of wrist or hand wire, rod, cerclage, or pin
NDJ70	Internal fixation of fracture of wrist or hand wire, rod, cerclage, or pin

Table S2. Annual incidence of distal radius fractures (DRF), specialist and primary care (with 95% confidence intervals).

	2015	2016	2017	2018	2019
Specialist care (95% Cl)	204,38 (200,59-208,15)	196,30 (192,60-200,00)	206,7,00 (203,21-210,80)	201,83 (198,10-205,57)	215,20 (211,34-219,06)
Primary care (95% CI)	85,83 (83,38-88,28)	71,57 (69,34-73,81)	75,46 (73,17-77,75)	67,80 (65,63-69,97)	47,74 (45,91-49,56)

Table S3. Incidence of DRF men and women in different age groups and years.

	2015		2016		2017		2018		2019	
**Age**	**Men**	**Women**	**Men**	**Women**	**Men**	**Women**	**Men**	**Women**	**Men**	**Women**
0-9	269,17	261,47	256,11	253,53	264,29	234,91	261,3	243,58	295,61	277,06
10-19	389,66	186,95	400,9	183,45	358,95	186,65	416,46	196,32	419,76	203,01
20-29	72,51	68,2	80,21	84,2	78,52	93,72	78,93	92,95	84,4	98,81
30-39	82,65	92,37	74,53	94,75	65,11	105,5	73,59	103,22	74,61	96,13
40-49	96,44	134,7	79,99	133,84	95,8	161,98	93,65	143,97	99,73	147,09
50-59	111,71	324,18	96,04	301,15	100,51	354,06	95,23	305,46	109,12	328,39
60-69	99,54	443,52	101,14	428,75	106,07	493,77	100,47	434,34	119,4	459,05
70-79	102,86	474,1	84,53	447,82	94,89	483,21	83,99	453,18	102,1	506,9
80-	116,12	512,47	124,8	470,37	132,18	470,98	124,01	518,35	103,8	543,35

Table S4. Annual incidence rate due to distal radius frac-ture (DRF) operative treatment in men and women.

	**Men**	**Women**
2015	30,02 (27,96-32,09)	53,99 (51,26-56,72)
2016	30,72 (28,63-32,80)	55,07 (52,32-57,52)
2017	30,19 (28,13-32,26)	62,40 (59,48-65,33)
2018	31,69 (29,58-33,80)	61,44 (58,54-64,35)
2019	33,07 (30,91-35,23)	60,38 (57,50-63,26)

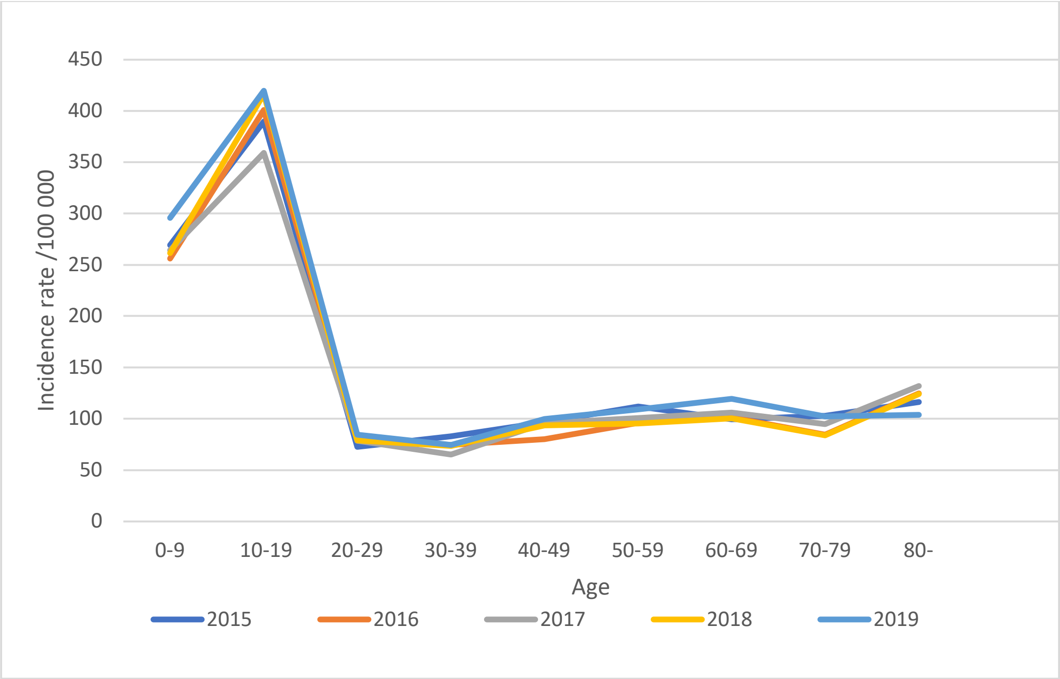

Figure S1. Annual incidence rate/100 000 in distal radius fractures in men different age groups years 2015-2019.

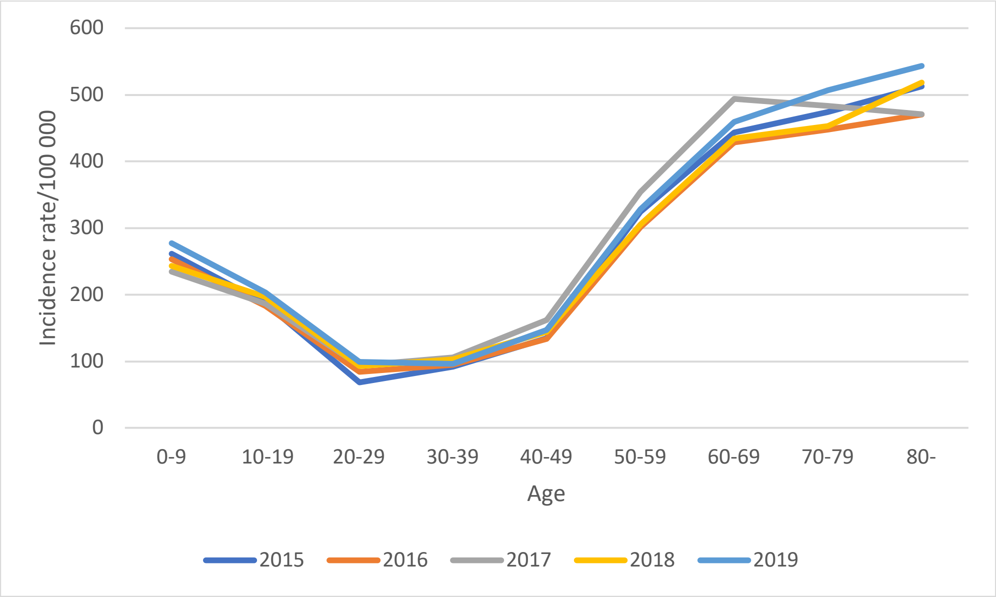

Figure S2. Annual incidence rate in distal radius fractures/100 000 in women in different age groups years 2015-2019.
